# Exploring the Syndecan-Mediated Cellular Internalization of the SARS-CoV-2 Omicron Variant

**DOI:** 10.3390/ijms241814140

**Published:** 2023-09-15

**Authors:** Annamária Letoha, Anett Hudák, Tamás Letoha

**Affiliations:** 1 Department of Medicine, Albert Szent-Györgyi Clinical Center, Faculty of Medicine, University of Szeged, 6720 Szeged, Hungary; letohadr@gmail.com; 2 Pharmacoidea Ltd., H-6726 Szeged, Hungary; anett.hudak@pharmacoidea.eu

**Keywords:** SARS-CoV-2, Delta variant, Omicron, cellular entry, endocytosis, syndecan, heparan sulfate proteoglycans

## Abstract

SARS-CoV-2 variants evolve to rely more on heparan sulfate (HS) for viral attachment and subsequent infection. In our earlier work, we demonstrated that the Delta variant’s spike protein binds more strongly to HS compared to WT SARS-CoV-2, leading to enhanced cell internalization via syndecans (SDCs), a family of transmembrane HS proteoglycans (HSPGs) facilitating the cellular entry of the original strain. Using our previously established ACE2- or SDC-overexpressing cellular models, we now compare the ACE2- and SDC-dependent cellular uptake of heat-inactivated WT SARS-CoV-2 with the Delta and Omicron variants. Internalization studies with inactivated virus particles showed that ACE2 overexpression could not compensate for the loss of HS in Omicron’s internalization, suggesting that this variant primarily uses HSPGs to enter cells. Although SDCs increased the internalization of all three viruses, subtle differences could be detected between their SDC isoform preferences. The Delta variant particularly benefitted from SDC1, 2, and 4 overexpression for cellular entry, while SDC4 had the most prominent effect on Omicron internalization. The SDC4 knockdown (KD) in Calu-3 cells reduced the cellular uptake of all three viruses, but the inhibition was the most pronounced for Omicron. The polyanionic heparin also hindered the cellular internalization of all three viruses with a dominant inhibitory effect on Omicron. Omicron’s predominant HSPG affinity, combined with its preference for the universally expressed SDC4, might account for its efficient transmission yet reduced pathogenicity.

## 1. Introduction

The significant global impact of the COVID-19 pandemic emphasizes the need for molecularly targeted, effective treatments against infections of the severe acute respiratory syndrome coronavirus 2 (SARS-CoV-2). Since its emergence, SARS-CoV-2 has undergone several mutations, resulting in different variants [1,2]. These variants have raised concerns due to their potential impacts on viral infectivity, transmissibility, and clinical outcomes. The Omicron variant, also known as B.1.1.529, was first identified in November 2021 in South Africa [3]. It quickly gained attention due to its large number of mutations, particularly in its spike protein, a region responsible for viral attachment and entry into host cells [4]. The Omicron variant possesses multiple mutations in its spike, raising concerns about its potential immune escape mechanisms [5,6,7]. The Omicron variant’s unique mutation profile suggests possible differences in its interaction with heparan sulfate (HS), which may have implications for viral infectivity and transmissibility [8,9].

HS is one of the main attachment sites for SARS-CoV-2 on the cell surface [10]. Multiple studies highlighted the significant role of HS in SARS-CoV-2 infection. [11,12]. The spike protein’s binding to HS prompts the “up/open” conformation necessary for the subsequent ACE2 binding [12]. Thus, HS acts as a co-receptor for SARS-CoV-2, amplifying the local viral concentration and aiding the virus’s spread throughout tissues [12,13]. The consistent presence of this mechanism among coronaviruses suggests that glycan attachment plays a pivotal role in SARS-CoV-2 infectivity [12].

Due to its trimeric structure, the spike protein offers multiple HS binding sites, allowing for versatile and multivalent spike-HS interactions [12,14,15]. Notably, mutations in the spike protein, especially in the Delta and Omicron variants, increase the positive charge in the presumed HS binding groove [9,16]. This suggests that these more infectious SARS-CoV-2 variants might have a stronger affinity for HS, facilitating their rapid spread [12,17].

Cell surface HS proteoglycans (HSPGs), such as glypicans and syndecans, play crucial roles in various processes of vertebrate cells, including cell adhesion, signaling, and development [18,19]. Glypicans are anchored to the cell membrane through glycosylphosphatidylinositol (GPI) and regulate the activity of growth factors [20]. Syndecans (SDCs), on the other hand, are transmembrane HSPGs made of a core protein, a transmembrane domain, and HS chains [21,22,23]. The four SDC members in humans exhibit cell-type-specific expression: SDC1 is predominantly found on epithelial cells; SDC2 is found on endothelial cells, fibroblasts, and some mesenchymal cell; SDC3 is primarily expressed by neurons [24,25,26,27]; and SDC4 is ubiquitously expressed but prominent in endothelial cells, fibroblasts, and epithelial cells, especially in the lung [28]. Due to their versatile HS chains, SDCs serve as binding sites for endogenous and exogenous ligands, including growth factors, cytokines, viruses, and other parasites [22,29]. Thus, SDCs can act as receptors or co-receptors for viral attachment and entry into host cells [30,31]. The interaction between viral envelope glycoproteins and the specific domains of SDCs mediates the binding and internalization of viruses, initiating the infection process [32,33].

Recent studies have highlighted the involvement of SDCs in SARS-CoV2 infections [34,35]. We have shown that SARS-CoV2 enters the cells after its spike protein attaches to SDCs on the cell surface [28,36]. Utilizing recombinant spike and spike-bearing pseudoviruses (PSVs), we have also shown the increased HS affinity of the Delta variant’s spike, along with the involvement of SDC1 and especially SDC4 in the spike protein’s cellular uptake [37]. Due to the rapid emergence of Delta and the hurry to explore potential antidotes, our studies on the Delta variant did not include the whole virus or all SDCs.

The Omicron variant has shown an even higher HS affinity than the Delta variant [9]. As the HS affinity of SARS-CoV-2 and its variants is a major contributor to viral pathogenesis, we conducted further research to fully elucidate the implications of Omicron’s increased HS affinity for host cell entry. Utilizing heat-inactivated viral particles, we compared the cellular internalization of the Omicron variant with the original Wuhan strain and the Delta variant in our previously established ACE2- and SDC-specific cellular models. Our comparative analyses led to new findings on Omicron’s unique viral entry properties arising from its increased HS affinity.

## 2. Results

### 2.1. Effect of ACE2 Overexpression on the Cellular Internalization of Inactivated Viruses

**Figure 1:**
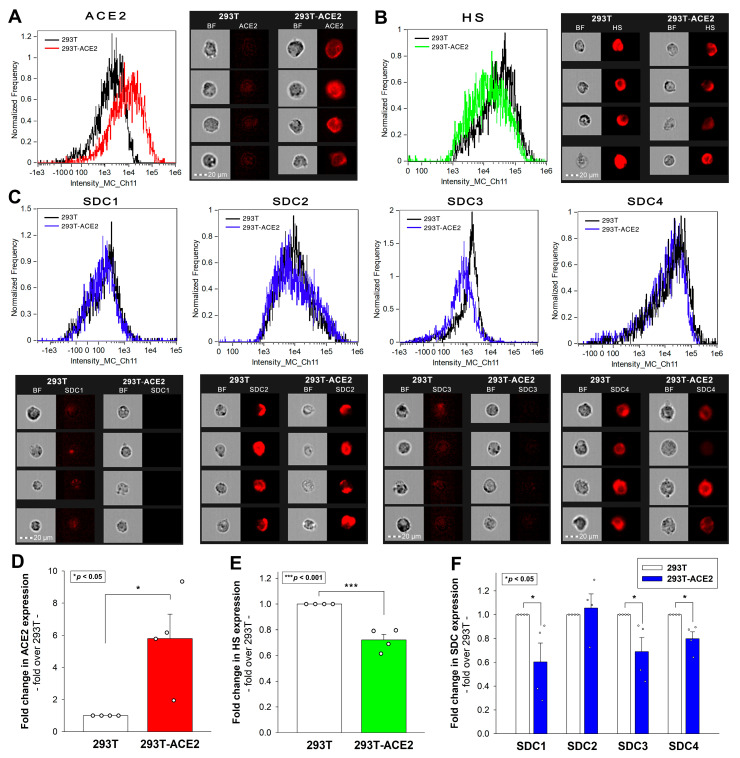
ACE2, HS, and SDC expression profiles of 293T and 293T-ACE2 cells. ACE2, HS, and SDC expression in 293T and 293T-ACE2 cells were assessed using fluorescently labeled specific antibodies with imaging flow cytometry. (**A**,**B**) ACE2 and HS expression profile of 293T and 293T-ACE2 cells. The representative flow cytometry histograms and cellular images show the ACE2 and HS expression of 293T and 293T-ACE2 cells treated with the fluorescent ACE2 and HS antibodies. Scale bar = 20 μm. (**C**) SDC expression profile of 293T and 293T-ACE2 cells. The representative flow cytometry histograms and fluorescent cellular images show the SDC expression of 293T cells and 293T-ACE2 cells treated with the APC-labeled respective SDC antibodies. Scale bar = 20 μm. (**D**–**F**) Detected ACE2, HS, and SDC expression values were normalized to those of 293T cells as standards. The bars represent the mean ± SEM of three independent experiments (data are represented as dots). Statistical significance vs. standards was assessed with ANOVA. * *p* < 0.05; *** *p* < 0.001.

The cell surface’s ACE2 receptor is commonly recognized as SARS-CoV-2’s main gateway for cellular entry [38,39]. To explore the involvement of ACE2 and HSPGs in the cellular uptake of the Omicron variant, we conducted comparative cellular uptake studies on previously established 293T-ACE2 cells overexpressing ACE2 (i.e., ACE2 expression of 293T-ACE2 cells is about 6× that of 293T cells) with a diminished (~70% that of 293T cells) HSPG expression (including SDCs, see Figure 1 and Supplementary Figure S1) [37]. The WT 293T cells and 293T-ACE2 cells were thus treated with either the heat-inactivated WT SARS-CoV-2 strain (WT SCV2) or the Delta or Omicron variants at 1 MOI for 4 h at 37 °C. Virus internalization was then detected by incubating the virus-treated, fixed, and permeabilized cells with an antibody specific to the spike’s amino acid sequence 1000–1200. To ensure that only intracellular viral particles were assessed, surface-attached viral particles were removed via trypsinization before the imaging flow cytometry analyses were conducted [28,37,40,41]. 

**Figure 2:**
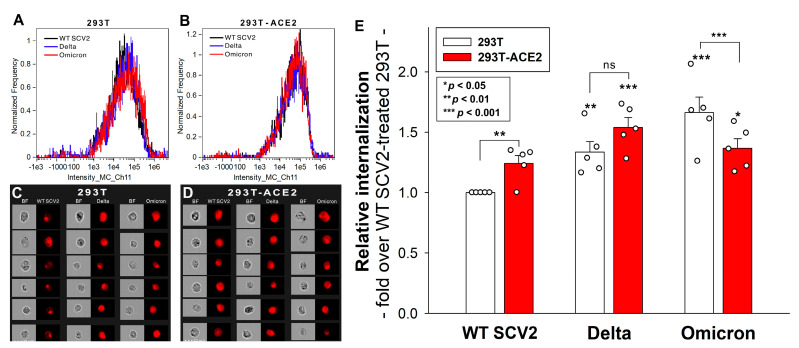
Cellular uptake of WT SCV2, Delta, and Omicron variants into 293T- and ACE2-overexpressing 293T-ACE2 cells. The cells were exposed to 1 MOI of heat-inactivated WT SCV2, Delta, and Omicron variants for 4 h at 37 °C. After incubation, the cells were washed, trypsinized, fixed, permeabilized, and treated with a primary SARS-CoV-2 spike (1000–1200 aa), followed by a fluorescently labeled (AF 633) secondary antibody. Cellular uptake was then analyzed with imaging flow cytometry. (**A**–**D**) Representative flow cytometry histograms and brightfield (BF) and fluorescent cellular images showing the intracellular fluorescence of the virus-exposed 293T and 293T-ACE2 cells. Scale bar = 20 μm. (**E**) Detected fluorescence intensities were normalized to WT SCV2-treated 293T cells as standards. The bars represent the mean + SEM of five independent experiments. Experimental data are presented as dots. Statistical significance was assessed with ANOVA. * *p* < 0.05; ** *p* < 0.01; *** *p* < 0.001; ns: not significant.

Compared to WT SCV2, both the Delta and Omicron variants were internalized more efficiently by the 293T cells (Figure 2A–E). A ~6-fold increase in the ACE2 expression with a ~30% reduced HS expression in the ACE2-293T cells (Figure 1B,E) resulted in a modestly (yet significantly, i.e., *p* < 0.01) increased WT SCV2 cellular uptake, an unchanged Delta cellular uptake, and a significantly reduced Omicron cellular uptake, highlighting the increased importance of HS in the variants, especially for Omicron’s cellular entry (Figure 2A–E).

Incubating the virus-exposed cells with secondary antibodies did not induce any differences in the fluorescence, showing that non-specific binding did not influence the obtained results (Supplementary Figure S2). Additionally, neither of the virus strains affected the cell viability at 1 MOI (Supplementary Figure S3).

### 2.2. SDCs Enhance the Cellular Uptake of All Three Viruses

**Figure 3:**
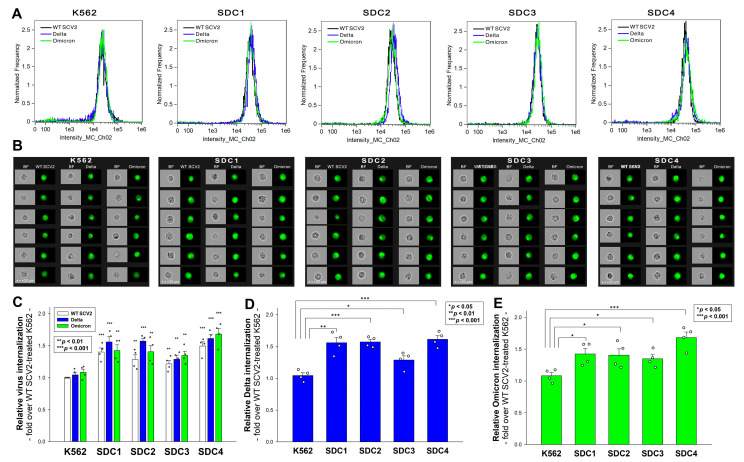
Cellular uptake of the heat-inactivated WT SCV2 and the Delta and Omicron variants into WT K562 cells and stable SDC transfectants. WT K562 cells and stable SDC transfectants were exposed to 1 MOI of the heat-inactivated WT SCV2 and the Delta and Omicron variants for 4 h at 37 °C. Cellular uptake was then analyzed with imaging flow cytometry using primary and fluorescently labeled secondary antibodies. (**A**,**B**) Representative flow cytometry histograms and cellular images showing the intracellular fluorescence of WT K562 cells and SDC transfectants treated with one of the viruses. Scale bar = 20 μm. (**C**–**E**) Detected fluorescence intensities normalized to WT SCV2-treated WT K562 cells as standards. The bars represent the mean + SEM of four independent experiments (data are represented as dots). Statistical significance vs. standards was assessed with ANOVA. * p < 0.05; ** p < 0.01; *** p < 0.001.

In our earlier research on the original Wuhan strain, we discovered that SDCs, particularly the ubiquitously expressed SDC4 that is found to be abundant in the lungs, facilitate WT SCV2’s cellular entry by interacting with the S1 subunit of its spike protein [28]. Numerous other studies have also emphasized the significance of SDC1 in WT SCV2 infection [35,42,43]. Later, by studying the Delta variant’s spike protein and the PSVs bearing the Delta spike, we showed that SDC1 and especially SDC4 enhance Delta’s cellular uptake. In the current study, we exposed our previously established stable SDC transfectant (created in WT K562 cells that are low in endogenous HSPG expression) to 1 MOI of either the heat-inactivated WT SCV2, Delta, or Omicron variant for 4 h at 37 °C. We must emphasize that the SDC transfectants utilized in our studies are standardized according to their HS expression (Supplementary Figure S4). Also, as noted in our previous report, SDC overexpression did not alter WT K562’s ACE2 expression (Supplementary Figure S5) [28,37]. Thus, the virus internalization studies were performed in SDC transfectants with even HS and ACE2 expressions using the above-described methodology. Imaging flow cytometry revealed that while SDC overexpression generally amplified the cellular entry of all viral particles, specific variations between the virus strains were observed (Figure 3A–E). The Delta variant showed the most pronounced increase in cellular entry in the cells overexpressing SDC1, 2, and 4, while the Omicron entered the SDC4 transfectants the most. This suggests that while the Delta variant exhibited evenly enhanced cellular uptake in all SDC transfectants, the Omicron variant predominantly favored SDC4 overexpression.

Incubating the cells with the AF 488-labeled secondary antibodies did not result in any statistically significant differences in the cellular fluorescence of applied WT K562 cells and SDC transfectants, showing that non-specific bindings did not influence the detected difference in the fluorescence intensities of the virus-treated cells (Supplementary Figure S6) [28]. The cellular viability studies showed that exposing the cells to 1 MOI for 4 h at 37 °C did not affect the cell viability (Supplementary Figure S7).

### 2.3. SDC4 Knockdown Affects Virus Internalization into Calu-3 Cells

**Figure 4:**
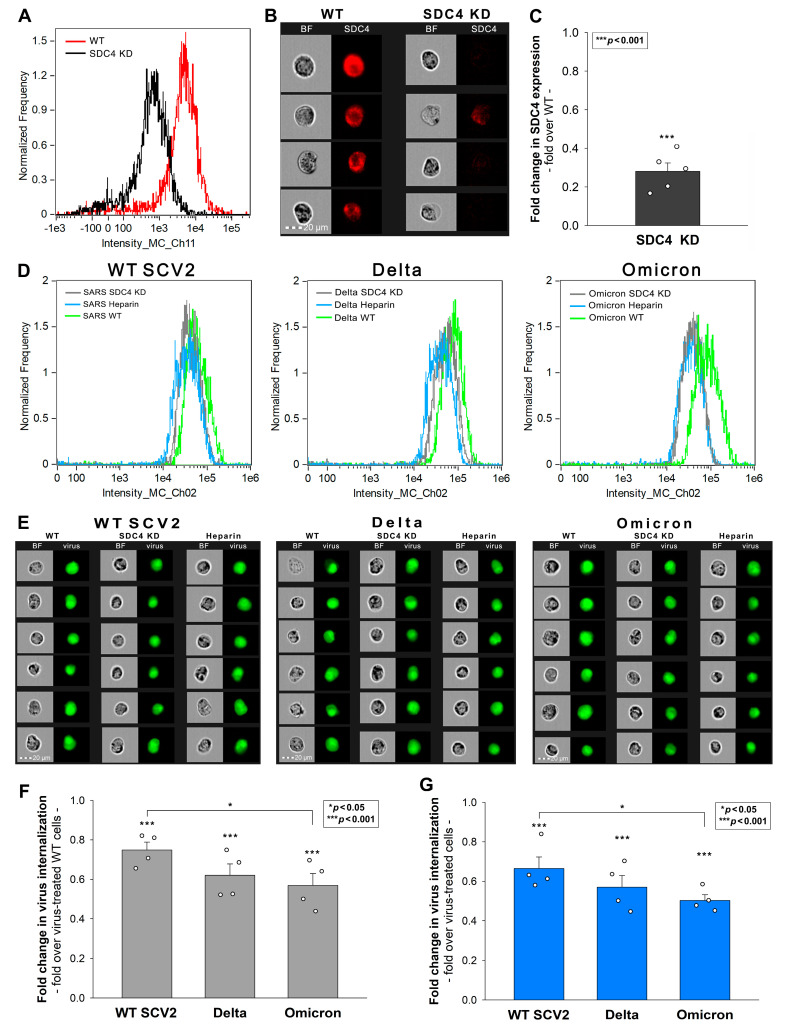
Effect of SDC4 knockdown (KD) or heparin inhibition on virus internalization into Calu-3 cells. SDC4 KD in Calu-3 cells was performed previously using a lentiviral vector specific to human SDC4. (**A**) SDC4 expression levels were measured with imaging flow cytometry, as shown by the representative histograms and cellular images. Detected SDC4 levels of KD cells were normalized to WT Calu-3 cells as standards. The bars represent the mean + SEM of four independent experiments. Statistical significance vs. standards was assessed with ANOVA. * *p* < 0.05. (**B**,**C**) SDC4 KD and WT Calu-3 cells were exposed to 1 MOI of the heat-inactivated WT SCV2, Delta, and Omicron variants. For GAG inhibition, the viruses were preincubated with heparin (200 ug/mL for 30 min at 37 °C) before being added to the cells. (**D**,**E**) Representative flow cytometry histograms and cellular images show the intracellular fluorescence of WT or SDC4 KD Calu-3 cells treated with the viruses in the presence or absence of heparin. Scale bar = 20 μm. (**F**,**G**) Detected intracellular fluorescent signals were normalized to WT Calu-3 (**F**) cells or cells untreated with heparin (**G**) as standards. The bars represent the mean + SEM of four independent experiments. Statistical significance vs. standards was assessed with ANOVA. * *p* < 0.05; *** *p* < 0.001.

Next, we examined the cellular uptake of the heat-inactivated WT strain and the Delta and Omicron variants in previously established SDC4 knockdown (KD) and WT Calu-3 cells (Supplementary Figures S8 and S9) [37]. After treating these cells with the respective viruses for 4 h, we analyzed their cellular uptake using imaging flow cytometry, as detailed above. Our findings reveal that SDC4 KD markedly diminished the cellular entry of all viruses (Figure 4A–G). Notably, the uptake of the Omicron variant was significantly lower compared to WT SCV2 in the SDC4 KD cells (Figure 4D–F). Compared with the WT Calu-3 cells, heparin inhibited the cellular entry of all the examined viruses, with the strongest effect observed for Omicron (Figure 4D,E,G). The cell viability studies showed that neither of the virus strains affected the cell viability at 1 MOI (Supplementary Figure S10).

**Figure 5:**
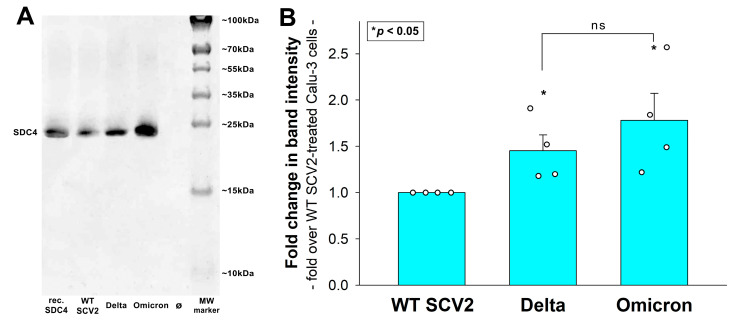
SDC4 binding of WT SCV2 and the Delta an Omicron variants. (**A**) SDS-PAGE showing SDC4 immunoprecipitated with an antibody specific for the spike’s amino acid sequence 1000-1200 from extracts of virus-treated Calu-3 cells. Lane 1: a total of 1 ug of recombinant SDC4; lanes 2–4: immunoprecipitates of Calu-3 cells treated with either WT SCV2, Delta, or Omicron, respectively; Lane 5: immunoprecipitate of untreated control Calu-3 cells. Standard protein size markers are indicated on the right. SDC4 signals were detected with UVITEC Alliance Q9 Advanced Imager, and the intensity of bands was analyzed with the NineAlliance© software. (**B**) Detected band intensities were normalized to WT SCV2-treated Calu-3 cells as standards. The bars represent the mean + SEM of four independent experiments. Statistical significance vs. standards was assessed with ANOVA. * p < 0.05; ns: not significant.

The co-immunoprecipitation (Co-IP) studies on the WT Calu-3 cells exposed to the viruses showed increased SDC4 binding of the Delta and Omicron variants compared to WT SCV2 (Figure 5A,B and Supplementary Figure S11). 

**Figure 6:**
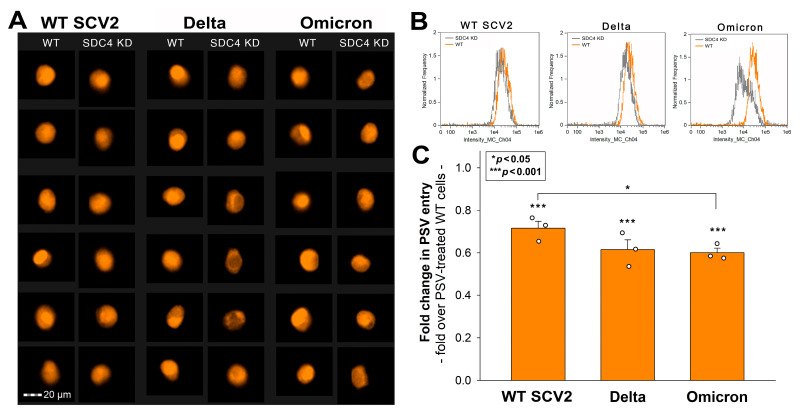
Effect of SDC4 KD on the cellular entry and gene delivery of WT SCV2, Delta, or Omicron PSVs in Calu-3 cells. SDC4 KD and WT Calu-3 cells were treated with either WT SCV2, Delta, or Omicron PSVs. (**A**,**B**) Representative cellular images and flow cytometry histograms showing the intracellular fluorescence of WT or SDC4 KD Calu-3 cells treated with the PSVs. Scale bar = 20 μm. (**C**) Detected fluorescence intensities were normalized to WT Calu-3 cells treated with the respective PSVs. The bars represent the mean + SEM of three independent experiments. Statistical significance vs. standards was assessed with ANOVA. * *p* < 0.05; *** *p* < 0.001.

The gene delivery studies with red fluorescent proteins (RFPs) encoding PSVs bearing the spike proteins of the respective strains (WT SCV2, Delta, or Omicron) showed that SDC4 KD decreased the cellular entry and gene delivery of all PSVs (Figure 6). Compared to the WT SCV2 spike-bearing PSVs, the Omicron spike-bearing PSVs exhibited significantly decreased gene delivery (*p* < 0.05; Figure 6C).

## 3. Discussion

SCV2 has undergone numerous mutations, leading to the emergence of various variants, including the Delta and Omicron variants [2,7]. The Omicron variant, in particular, has garnered significant attention due to its extensive mutation profile, with more than 60 genetic mutations compared to the wild type [44]. Studies have indicated that these mutations increase viral fitness and infectivity [45]. The Delta variant, which was prevalent before the emergence of Omicron, also exhibited enhanced infectivity compared to the WT SCV2 [17]. Understanding the interactions between the virus and host cells is crucial for developing effective treatment strategies.

We previously explored the interaction of WT SCV2 viral particles and the spike proteins of WT SCV2 and the Delta variant with SDCs and ACE2 [28,37]. Our findings indicate that both WT SCV2 and Delta spikes attach to SDCs and get internalized by human cells via an SDC-mediated uptake. We also showed that an SDC-mediated uptake gains importance for Delta, a variant with increased net positive charges in its spike protein. However, our exploration of the Delta variant was limited to spike proteins and spike-bearing PSVs. Our studies with the Delta spike proteins only included SDC1 and 4, the two SDC isoforms with reportedly dominant roles in WT SCV2 uptake. Studies with the Delta spike highlighted the increased dependency on HSPGs and SDCs for cell entry, especially emphasizing the important role of SDC4.

Considering the increased HS affinity of the Omicron variant, we pursued comparative cellular uptake studies with WT SCV2 and the Delta and Omicron variants. Our analysis of heat-inactivated virus particles in ACE2- and SDC-specific cell models revealed Omicron’s stronger preference for HSPG over ACE2. ACE2 overexpression with a reduced HSPG expression resulted in a modest (~24%) yet significantly increased WT SCV2 uptake, an unchanged Delta entry, and a notable decrease (~17%) in Omicron uptake. These results correlate with the altered HS affinity of the variants: WT SCV2 < Delta < Omicron. Thus, Omicron, possessing the highest net positive charges, predominantly favors an attachment to polyanionic HS and enters cells facilitated by HSPGs. Compared to Omicron, the decrease in HSPG expression had a smaller effect on WT SCV2, with fewer net positive charges. The finding that a ~5.8-fold increase in ACE2 expression, combined with a ~30% reduction in HS, only led to a modest ~24% growth in WT SCV2′s uptake, underscores the significant role of HSPGs in the cellular entry of the original Wuhan strain.

Subsequent investigations on stable SDC transfectants unveiled nuanced differences in the SDC isoform affinities among the variants. While the SDC overexpression increased the cellular entry of all studied strains, there were variations. SDC1, 2, and 4 increased Delta’s entry, while Omicron’s uptake was the highest in SDC4 transfectants. The even HS expression of our SDC transfectants suggests that factors beyond the HS chains also contribute to the SDC preferences of the variants. The SDC4 knockdown (KD) and heparin co-treatment decreased the viral uptake in Calu-3 cells, with Omicron being the most affected. The co-immunoprecipitation (Co-IP) studies further confirmed Delta’s and Omicron’s enhanced binding to SDC4.

Our in vitro investigations demonstrate Omicron’s pronounced reliance on HSPG for cell entry, with a particular preference for the ubiquitously expressed SDC4 isoform. On the other hand, Delta spreads its affinity across SDC1, 2, and 4. The preference for HSPG-mediated uptake and the dominant role of SDC4 vs. different SDC isoforms might contribute to Omicron’s increased transmissibility yet reduced pathogenicity. These attributes potentially render Omicron susceptible to heparin-based therapeutics. To ascertain the therapeutic implications of our findings, further experiments with virulent strains in clinically relevant in vivo settings are imperative. 

## 4. Materials and Methods

### 4.1. Heat-Inactivated Virus Strains and PSVs

Heat-inactivated WT SCV2 (strain: 2019-nCoV/USA-WA1/2020), Delta (strain: USA/MD-HP05285/2021), and Omicron (strain: USA/COR-22-063113/2022) variants were purchased from ATCC (Manassas, VA, USA; cat. nos. ATCC VR-1986HK, VR-3342HK, and VR-3342HK, respectively). 

RFPs encoding PSVs, recombinant pseudotyped lentiviral particles bearing the spike of the respective virus strains (WT SCV2, Delta, or Omicron), and encoding RFPs were purchased from GeneMedi (Shanghai, China; cat. nos. GM-2019nCoV-PSV01, GM-2019nCoV-PSV34, and GM-2019nCoV-PSV40, respectively).

### 4.2. ACE2 and SDC Constructs, Cell Culture, and Transfection

The stable transfectants of human ACE2 established in 293T cells (i.e., 293T-ACE2 cells) and its parent 293T cell line were purchased from GeneMedi (Shanghai, China; cat. no. GM-SC-293T-hACE2-01) and Merck KGaA (Darmstadt, Germany; cat. no. 12022001). SDC transfectants, established in K562 cells (ATCC CCL-243), were created as described previously [28,29]. 

### 4.3. Flow Cytometry Analysis of HS an ACE2 and SDC Expression

HS and SDC expression of the applied cell lines (293T, 293T-ACE2, K562, and SDC transfectants) were measured with flow cytometry by using anti-human HS antibody (10E4 epitope; Amsbio, Abingdon, UK; Alexa Fluor (AF) 647-labeled secondary anti-mouse IgM and respective isotype control, Thermo Fisher Scientific, Waltham, MA, USA, cat. no. 02-6800) and APC-labeled SDC antibodies as described previously [29,46]. SDC transfectants (created in K562 cells) with almost equal amounts of HS expression were selected for further uptake studies [29,46]. ACE2 expression was measured with human ACE2 AF 647-conjugated antibody (RnD Systems, Minneapolis, MN, USA, cat. no. FAB9332R) and respective isotype control (mouse IgG2A AF 647-conjugated isotype control, RnD Systems, cat. no. IC003R), according to the manufacturer’s protocol.

### 4.4. Creation of SDC4 KD Cell Lines

SDC4 knockdown in Calu-3 cells was performed as described previously, using a lentiviral vector system specific to human SDC4 shRNA (cat. nos. sc-41400-SH an sc-36588), according to the manufacturer’s protocol (Santa Cruz Biotechnology, Inc., Dallas, TX, USA) [37]. Stable KD cells were selected in 2 mg G418 and sorted using imaging flow cytometry (Amnis FlowSight, Luminex Corporation, Austin, TX, USA) with APC-conjugated anti-SDC4 antibody (RnD Systems, Minneapolis, MN, USA, cat. no. FAB29181A) and respective isotype control (rat IgG2A APC isotype control, RnD Systems, cat. no. IC006A). The cellular expression of SDC4 following knockdown was determined with Western blotting as described previously [37]. 

### 4.5. Flow Cytometry Analysis of Virus Uptake

293T and 293T-ACE2 cells, K562 and its SDC transfectants, along with WT and SDC4 KD Calu-3 cells were utilized to quantify the internalization of the virus strains. Briefly, 3 × 10^5^ cells/mL in DMEM medium were exposed to 1 MOI of one of the SCV2 strains (WT, Delta, or Omicron) for 4 h at 37 °C. After 4 h of incubation, the cells were washed and trypsinized (using the method described by Nakase et al. [28,40,41]) to remove the extracellularly attached viruses from the cell surface. The cells were then washed, fixed, permeabilized, and treated with mouse monoclonal (1A9) antibody specific to SCV2 spike glycoprotein amino acid sequence 1000–1200 (Abcam, Cambridge, UK, cat. no. 273433). After incubation for 1 h at room temperature, the cells were treated for 1 h at room temperature with either AF 488- (WT K562 cells and SDC transfectants, WT or SDC4 KD Calu-3) or AF 633-labeled (293T and 293T-ACE2 cells) goat anti-mouse IgG (both Invitrogen, Carlsbad, CA, USA, cat. no. A-11001 and A-21052, respectively). The samples were then rinsed three times with PBS containing 1% BSA and 0.1% Triton X-100 and progressed toward flow cytometry. Cellular uptake was then measured with flow cytometry using an Amnis FlowSight imaging flow cytometer (Amnis Corporation, Seattle, WA, USA). A minimum of 5000 events per sample were analyzed. Appropriate gating in a forward-scatter-against-side-scatter plot was utilized to exclude cellular debris and aggregates. Fluorescence analysis was conducted with the Amnis IDEAS analysis software (Amnis Corporation, Seattle, WA, USA).

### 4.6. Cell Viability Measurements

The effect of the applied heat-inactivated viral particles on cell viability was assessed with the EZ4U cell proliferation assay (Biomedica Gmbh, Vienna, Austria, cat. no. BI-5000) according to the manufacturer’s instructions. Absorbance was measured with a BioTek Cytation 3 multimode microplate reader.

### 4.7. Co-Immunoprecipitation Experiments

WT Calu-3 cells exposed to 1 MOI of the heat-inactivated viral particles were processed for Co-IP experiments as described previously [28,29,46]. After incubation, the cells were washed twice with ice-cold PBS and treated with a cold Pierce IP lysis buffer. The cells were then scraped off to clean Eppendorf tubes, put on a low-speed rotating shaker for 15 min, and centrifuged at 14,000× *g* for 15 min at 4 °C. The supernatant was transferred to new tubes and combined with a 5 µg mouse monoclonal (1A9) antibody specific to SCV2 spike glycoprotein (Abcam, Cambridge, UK, cat. no. 273433). The antigen sample/SDC antibody mixture was then incubated overnight at 4 °C with mixing. The antigen sample/antibody mixture was then added to a 1.5 mL microcentrifuge tube containing pre-washed Pierce Protein A/G Magnetic Beads (Thermo Fisher Scientific, Waltham, MA, USA). After incubation at room temperature for 1 h with mixing, the beads were collected with a MagJET Separation Rack magnetic stand (Thermo Fisher Scientific, Waltham, MA, USA), and supernatants were discarded. An amount of 100 µL of SDS-PAGE reducing sample buffer was added to the tubes to elute the antigen. The samples were heated at 96 °C for 10 min in 1% SDS and transferred to SDS-PAGE [28,29,46]. The samples were then immunoblotted onto PVDF membranes, and SDC4 proteins were detected with specific human SDC4 antibodies (5G9, Santa Cruz Biotechnology, Inc., Dallas, TX, USA; cat. no. sc-12766) and anti-mouse IgG-HRP secondary antibody (Invitrogen, Carlsbad, CA, USA; cat. no. 31450). Image acquisition was conducted with the UVITEC Alliance Q9 Advanced imaging platform (Uvitec Ltd., Cambridge, UK). Band intensities were analyzed with the NineAlliance© software (Uvitec Ltd., Cambridge, UK).

### 4.8. PSV Studies

WT and SDC4 KD Calu-3 cells were seeded in 24-well plates with 1 × 10^5^ cells/well. After 24 h of culture, cells were treated with 2 × 10^6^ transducing units of spike-protein-bearing PSV-RFPs according to the manufacturer’s instructions (GeneMedi, Shanghai, China). After 72 h of incubation, RFP expression of PSV-treated cells was assessed with imaging flow cytometry (Amnis FlowSight, Luminex, Austin, TX, USA).

### 4.9. Statistical Analysis

Results are expressed as means + standard error of the mean (SEM). Differences between experimental groups were evaluated using one-way analysis of variance (ANOVA). Values of *p* < 0.05 were accepted as significant [28,29,46].

## Data Availability

Data are contained within the article or Supplementary Materials.

## Supplementary Materials

The following are available online at https://www.mdpi.com/article/10.3390/ijms241814140/s1.
