# Supplementary material for: Exploring the Syndecan-Mediated Cellular Internalization of the SARS-CoV-2 Omicron Variant
Source: Int J Mol Sci. 2023 Sep 15;24(18):14140. doi: 10.3390/ijms241814140 (PMC10531417; doi:10.3390/ijms241814140)
Supplement: Supplementary file 1 [file ijms-24-14140-s001.zip › ijms-2584859-supplementary.pdf]

Supplementary Materials: Exploring the syndecan-mediated cellular internalization of the SARS-CoV-2 Omicron variant

Annamária Letoha, Anett Hudák, and Tamás Letoha

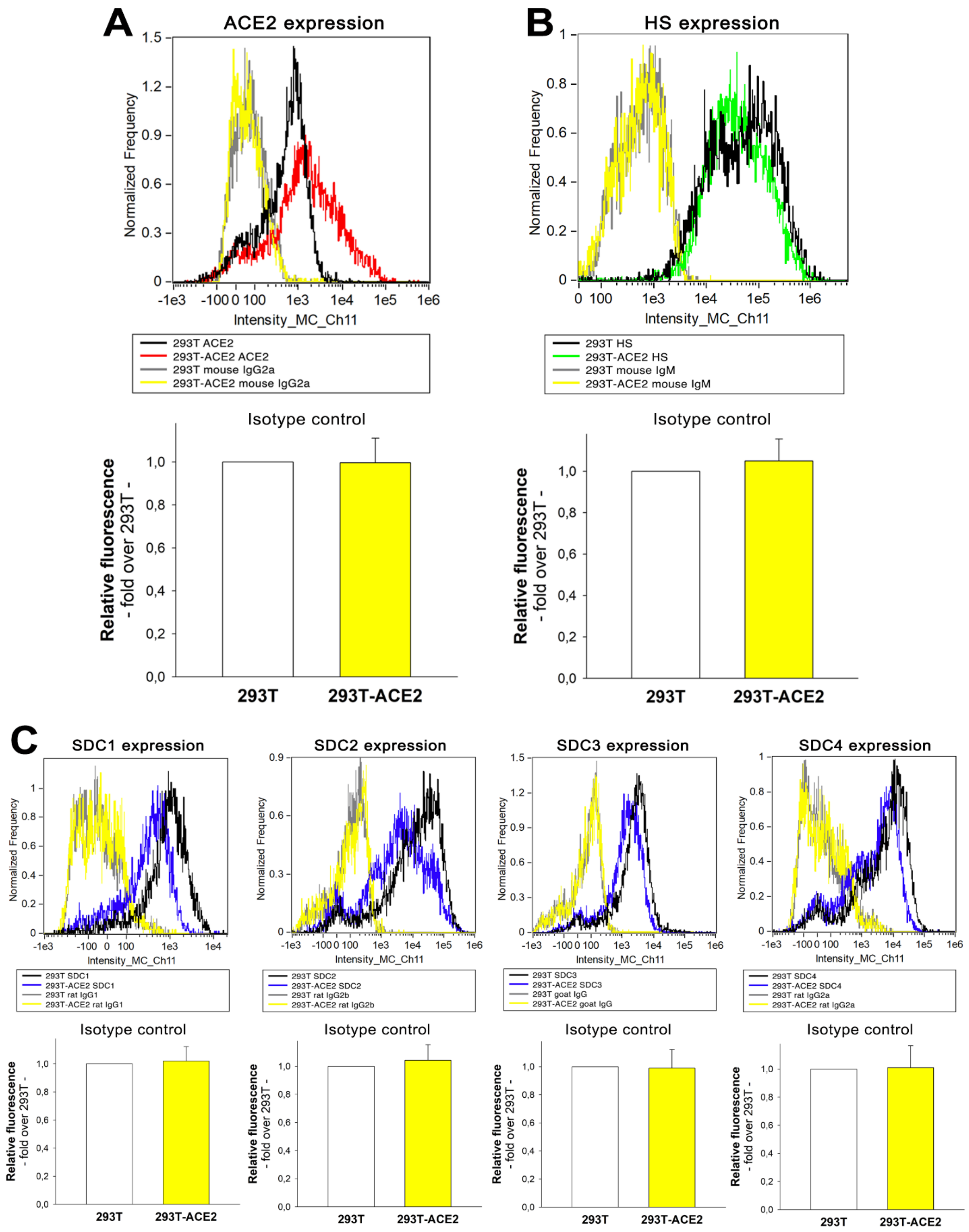

Figure S1. Control studies with 293T and 293T-ACE2 cells treated with ACE2, HS, or SDC antibodies and respective isotype controls. The cells were treated with the appropriate antibodies and respective isotype controls. Cellular fluorescence was then measured with flow cytometry. **(A-C)** Flow cytometry histograms showing cellular fluorescence of cells treated with anti-ACE2 (**A**), anti-HS (**B**), anti-SDC (**C**) antibodies, or respective isotype controls. Detected fluorescence intensities of isotype control-treated cells were normalized to isotype control-treated 293T cells as standards. The bars represent the mean + SEM of three independent experiments. Statistical significance vs. standards was assessed with ANOVA. Analyses did not exhibit statistically significant differences in cellular fluorescence of isotype-treated cells.

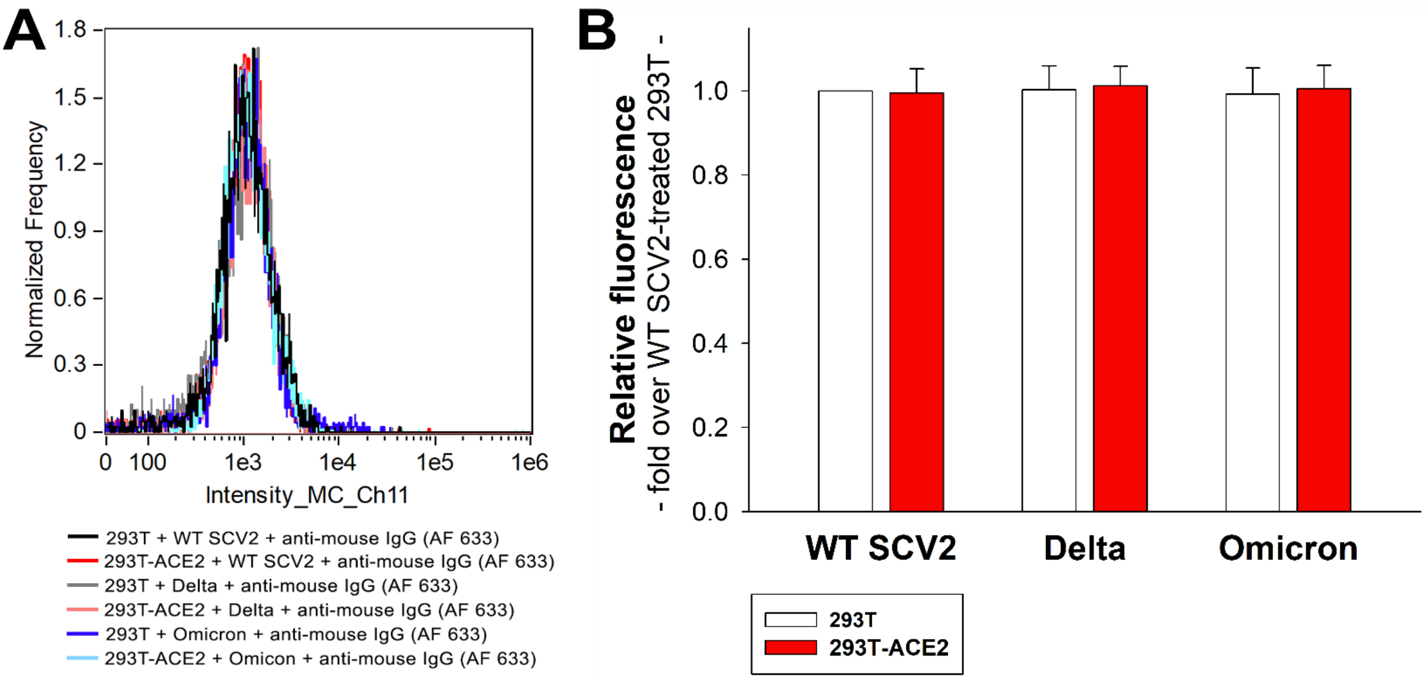

Figure S2. Cellular fluorescence of virus-exposed 293T and 293T-ACE2 cells after AF 633-labeled secondary antibody treatment. The cells were exposed to 1 MOI of heat-inactivated WT SCV2, Delta, and Omicron viruses for 4 h at 37 °C. After incubation, the cells were washed, trypsinized, fixed, permeabilized, and treated with fluorescently labeled (AF 633) secondary antibodies. Fluorescence was then analyzed with imaging flow cytometry. (A) Representative flow cytometry histograms showing the intracellular fluorescence of the virus-exposed 293T and 293T-ACE2 cells. (B) Detected fluorescence intensities were normalized to WT SCV2-treated 293T cells as standards. The bars represent the mean + SEM of five independent experiments. Experimental data are presented as dots. Statistical significance was assessed with ANOVA. No statistically significant differences were detected between the studied cell lines.

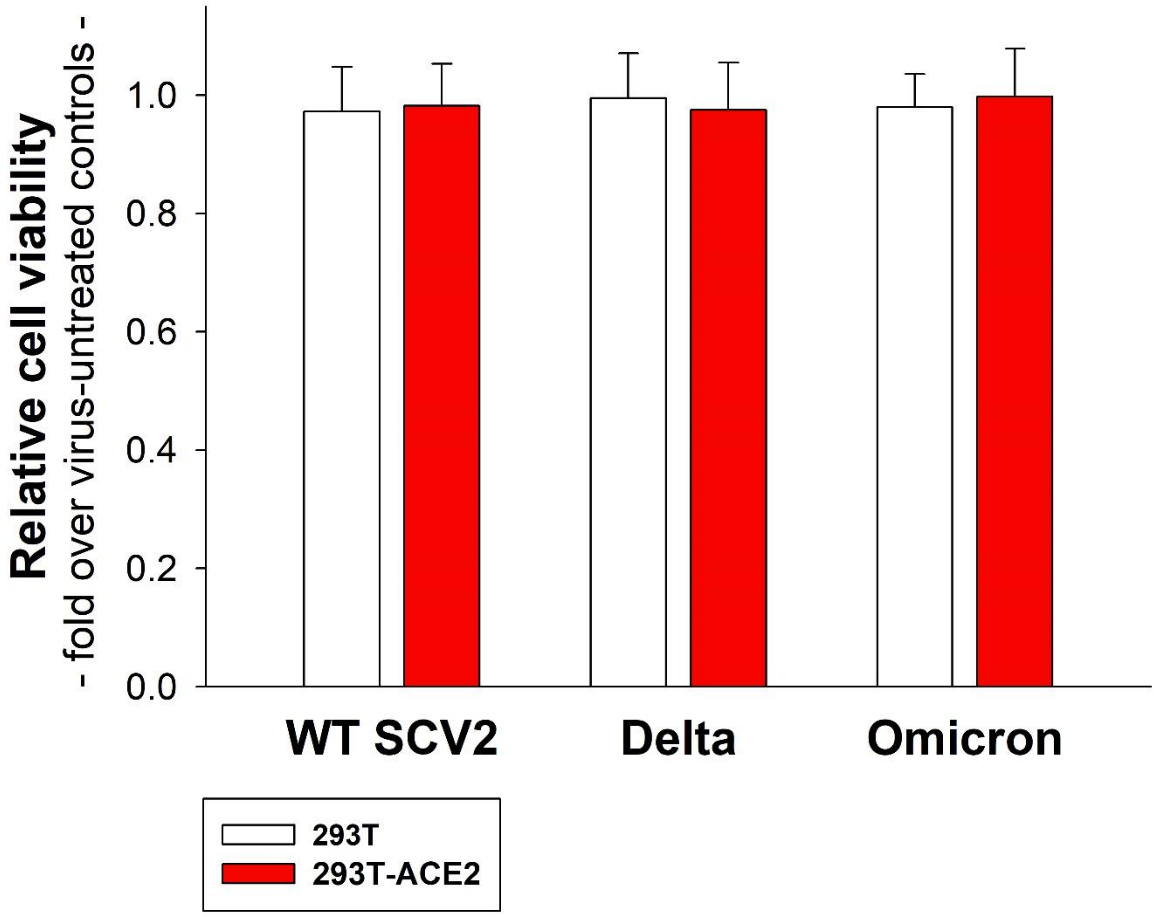

Figure S3. Heat-inactivated WT SCV2, Delta, and Omicron viruses do not affect the cellular viability of 293T and 293T-ACE2 cells at 1 MOI. 293T and 293T-ACE2 cells treated with or without WT SCV2, Delta, or Omicron for 4 h at 1 MOI at 37 °C. Cellular viability was then measured with EZ4U assay and detected measures were then normalized to untreated cells as controls. The bars represent the mean + SEM of five independent experiments. Statistical significance vs. controls was assessed with ANOVA. Compared to controls, no statistically significant differences were detected in the viability of virus-treated cells.

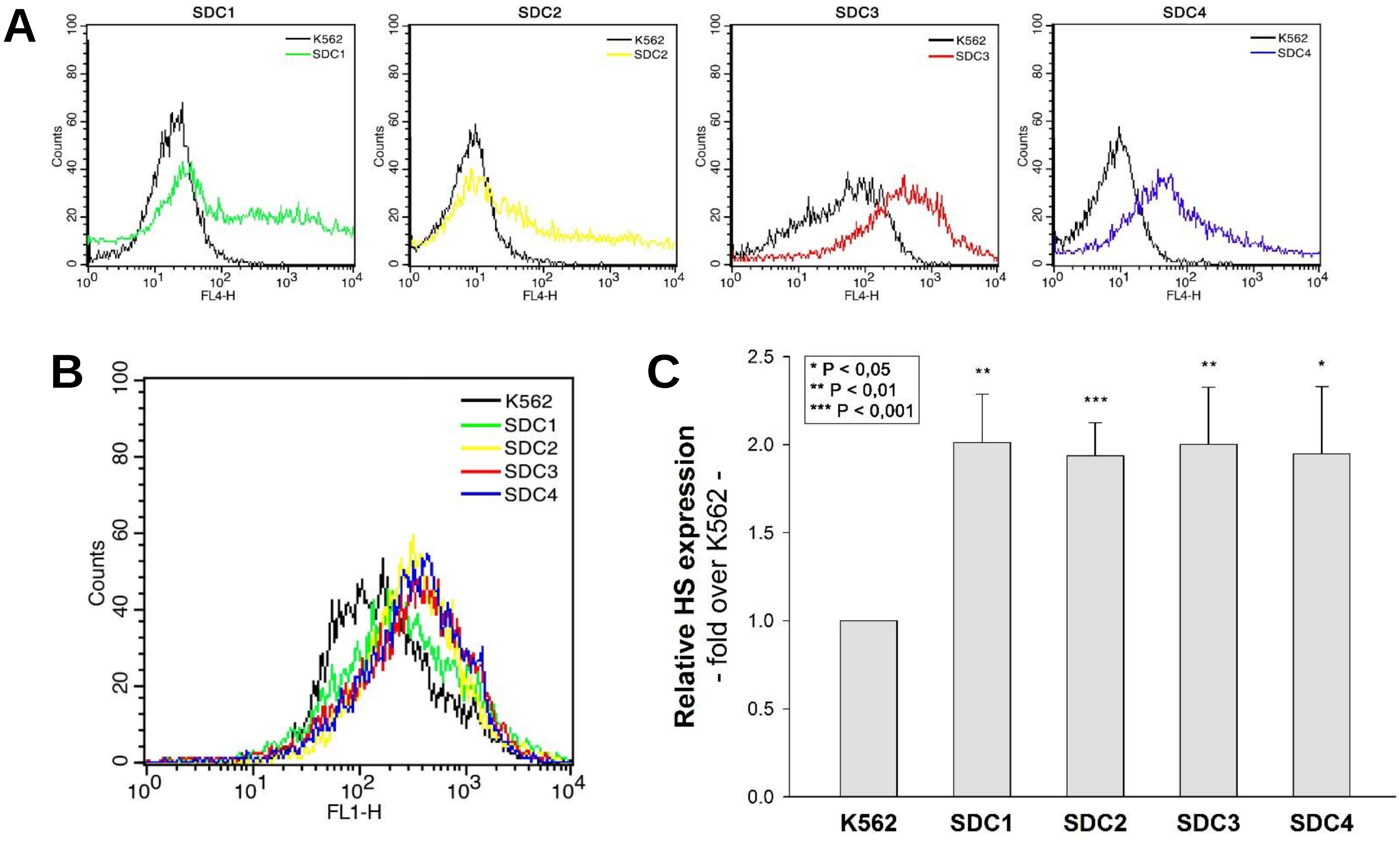

Figure S4. Relative HS expression of SDC transfectants. (**A**) Stable SDC transfectants created in wild-type (WT) K562 cells were selected by measuring SDC expression with flow cytometry (Becton Dickinson FACScan) using APC-labeled anti-SDC antibodies specific for each SDC isoform. (**B**) Flow cytometry histograms showing HS expression of SDC transfectants and WT K562 cells. HS expression of SDC transfectants, along with WT K562 cells, was measured by flow cytometry (Becton Dickinson FACScan) using an anti-HS antibody. SDC transfectants with similar HS expression were selected and applied for further studies. (**C**) Detected HS levels were normalized to WT K562 cells as standards. The bars represent the mean + SEM of ten independent experiments. Statistical significance vs. WT K562 cells (standards) was assessed with analysis of variance (ANOVA). *$p<0.05$ vs WT K562 cells; **$p<0.01$ vs WT K562 cells, ***$p<0.001$ vs WT K562 cells.

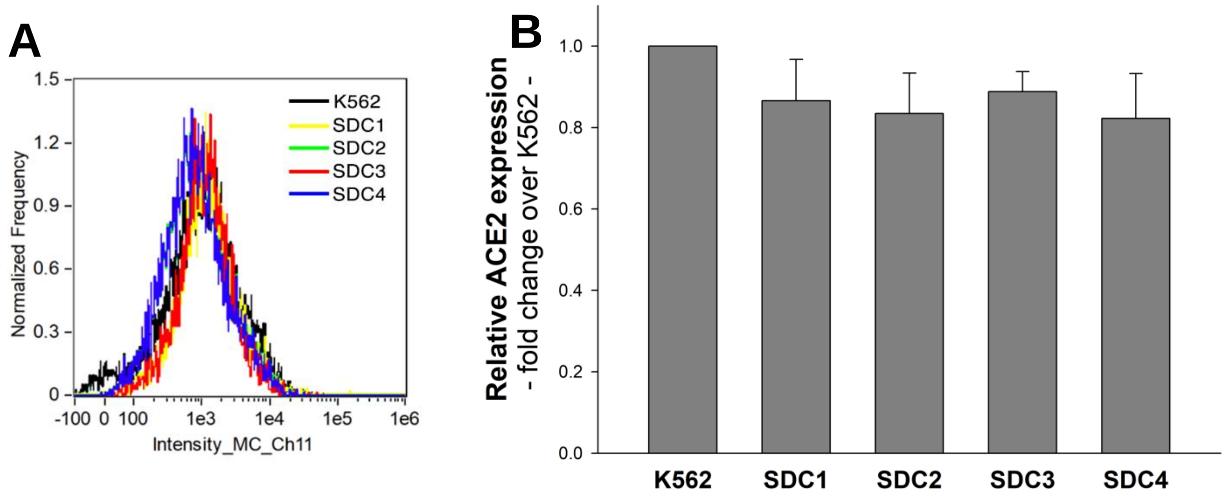

Figure S5. Relative ACE2 expression of K562 cells and SDC transfectants. ACE2 expression of WT K562 and SDC transfectants (created in K562 cells) was measured with flow cytometry using AF 647-labeled ACE2 antibody. (**A**) Representative flow cytometry histograms showing the ACE2 expression of SDC transfectants and WT K562 cells. (**B**) Detected ACE2 levels were normalized to WT K562 cells as standards. The bars represent the mean + SEM of three independent experiments. Statistical significance vs. WT K562 cells as standards was assessed with analysis of variance (ANOVA). Compared to WT K562 cells, no statistically significant differences were detected in ACE2 expression of SDC transfectants.

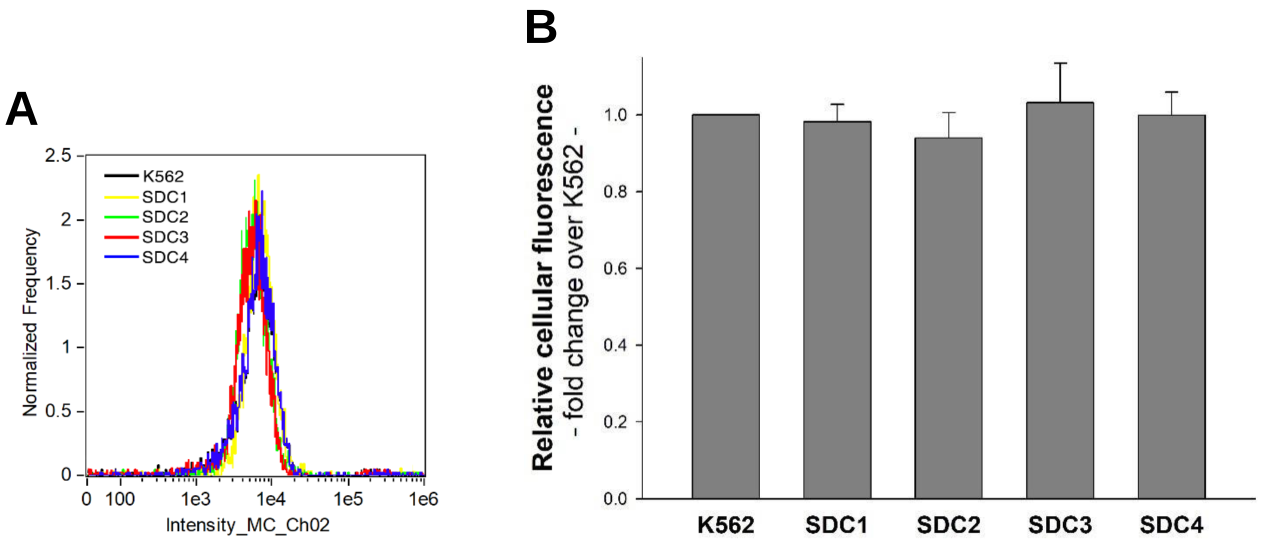

Figure S6. Cellular fluorescence of WT K562 cells and SDC transfectants after AF 488-labeled secondary antibody treatment. WT K562 cells and SDC transfectants were rinsed, fixed, permeabilized, and blocked with the appropriate goat serum for 1h at room temperature, followed by AF 488-labeled secondary antibody treatment. After 1 h of incubation, the fluorescence of cells was measured with flow cytometry. (**A**) Representative flow cytometry histograms showing the fluorescence of WT K562 cells and SDC transfectants incubated with AF 488-labeled goat anti-mouse secondary antibodies. (**B**) Detected fluorescence intensities were normalized to antibody-treated WT K562 cells as standards. The bars represent the mean + SEM of three independent experiments. Statistical significance vs. standards (WT K562 cells) was assessed with ANOVA. Compared to WT K562 cells, no statistically significant differences were detected.

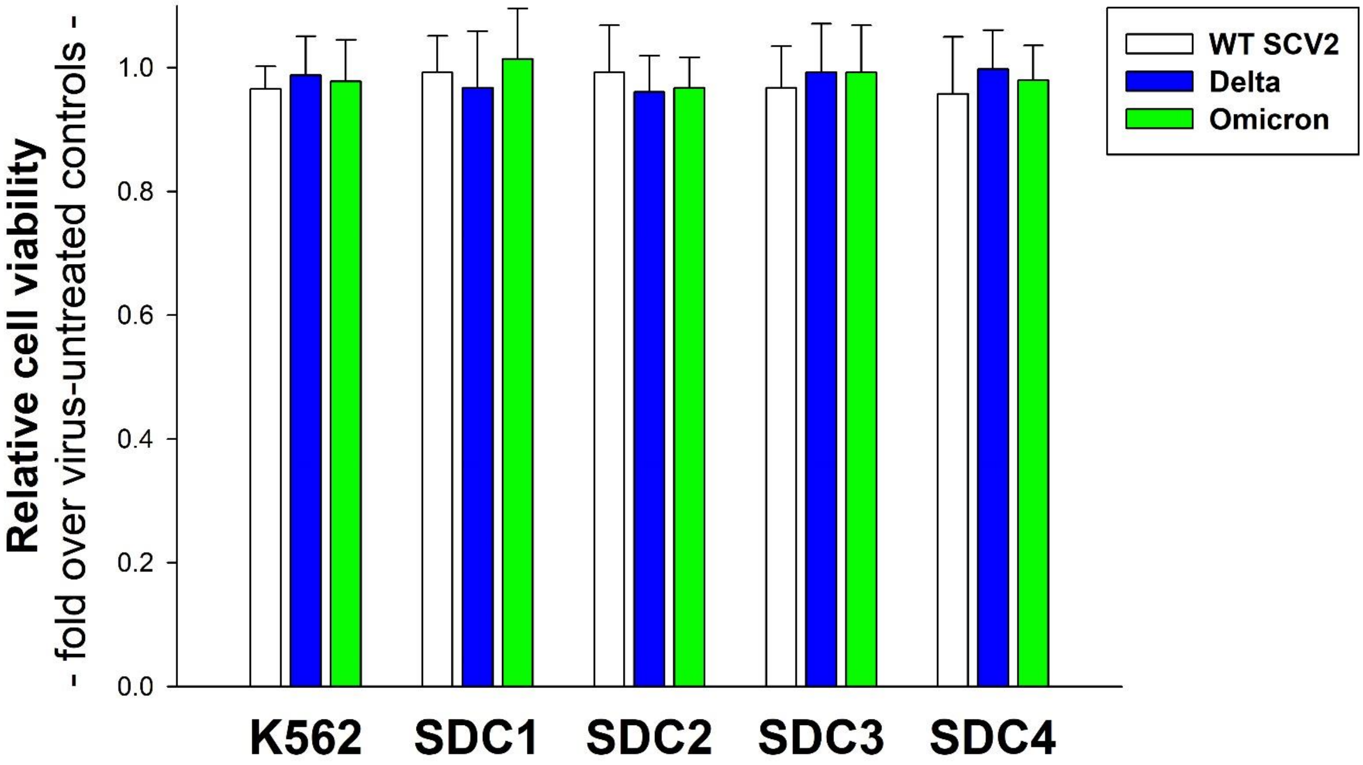

Figure S7. Heat-inactivated WT SCV2, Delta, and Omicron variants do not affect the cellular viability of WT K562 cells SDC transfectants after 4 h incubation at 1 MOI. WT K562 cells and SDC transfectants were incubated with either WT SCV2, Delta, or Omicron variants for 4 h at 1 MOI at 37 °C. Cellular viability was then measured with EZ4U assay and detected measures were then normalized to untreated cells as controls. The bars represent the mean + SEM of three independent experiments. Statistical significance vs. controls was assessed with ANOVA. Compared to controls, no statistically significant differences were detected in the viability of virus-treated cells.

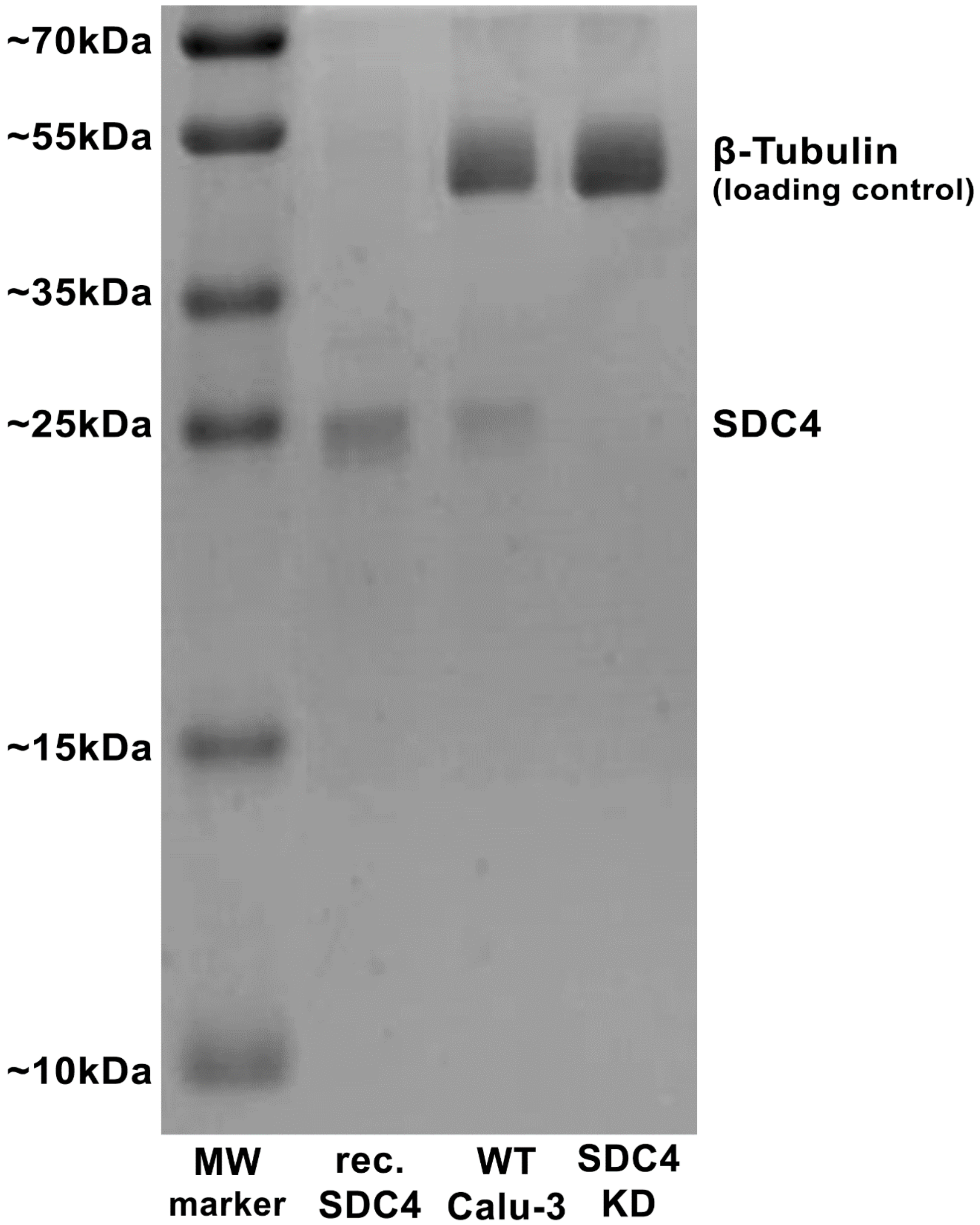

Figure S8. Western blot validation of SDC4 knockdown in Calu-3 cells. SDC4 knockdown (KD) in Calu-3 cells was performed using a lentiviral vector system specific to human SDC4 shRNA. Stable KD cells were selected in 2 mg G418 and sorted using imaging flow cytometry (Amnis FlowSight) with APC-conjugated anti-SDC4 antibodies. Cellular expression of SDC4 following knockdown was also determined with Western blotting. WT Calu-3 and SDC4 shRNA-treated cells were grown in 24-well plates for 24 h, then the medium was removed, and the cells were washed (with PBS) and lysed in RIPA buffer. Protein concentrations were measured with a spectrophotometer (Metertech UV/VIS). Equal amounts of protein from cell lysates were then subjected to SDS-PAGE on 7.5–12.5% gradient gels and electroblotted onto PVDF membranes using the Mini Wide Vertical Electrophoresis gel system (Cleaver Scientific). The membranes were blocked in TBST with 5% dry milk, washed, incubated with anti-SDC4 antibodies diluted in TBST with 1% dry milk for 2 h, and then incubated with HRP-conjugated secondary antibodies. A chemiluminescence detection reagent was used for protein visualization, and the signal was detected with UVITEC Alliance Q9 Advanced Imager. β-Tubulin was used as a loading control.

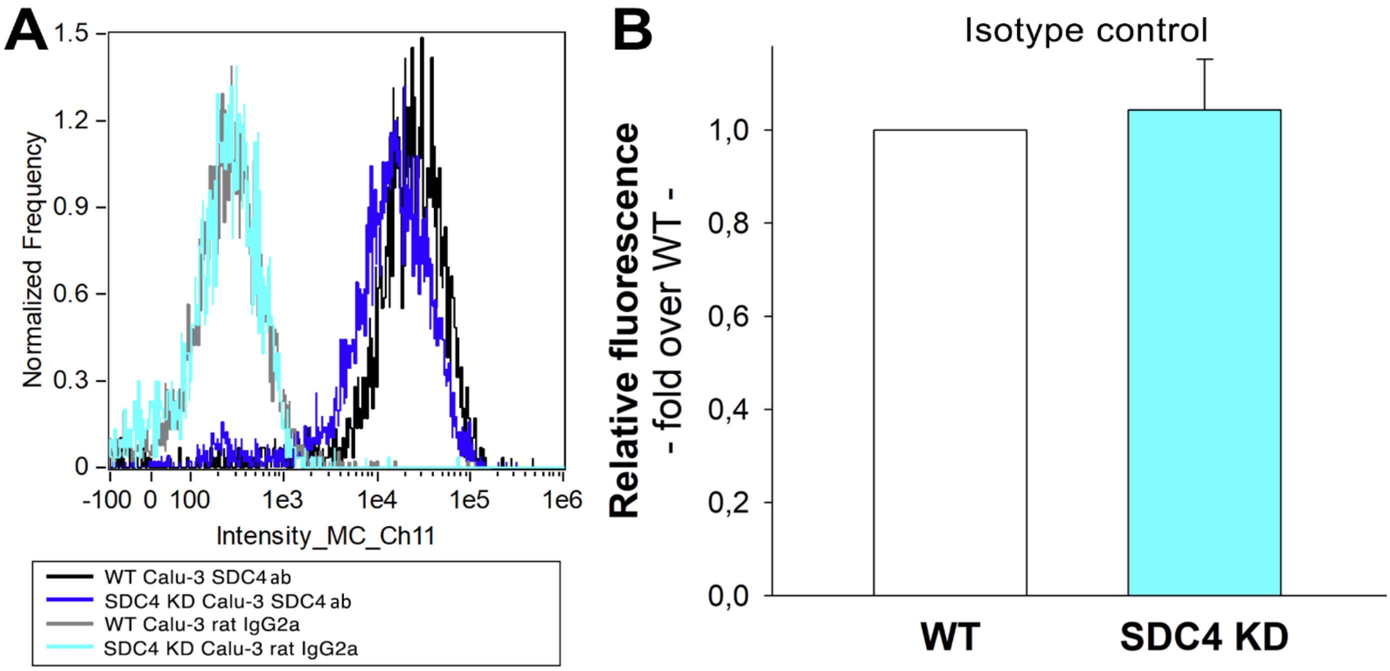

Figure S9. Control studies with WT or SDC4 KD Calu-3 cells treated with APC-labeled SDC4 antibody or the respective isotype control. WT or SDC4 KD Calu-3 cells were treated with APC-labeled SDC4 antibody or the respective isotype control for 1 h. After incubation with the antibodies, the cells were washed, and cellular fluorescence was then measured with flow cytometry. (**A**) Flow cytometry histograms showing cellular fluorescence of WT or SDC4 KD Calu-3 cells treated with a-SDC4 antibody and respective isotype control. (**B**) Detected fluorescence intensities normalized to WT Calu-3 cells treated with the isotype control. The bars represent the mean + SEM of three independent experiments. Statistical significance vs. standards was assessed with ANOVA. No statistical significance was detected.

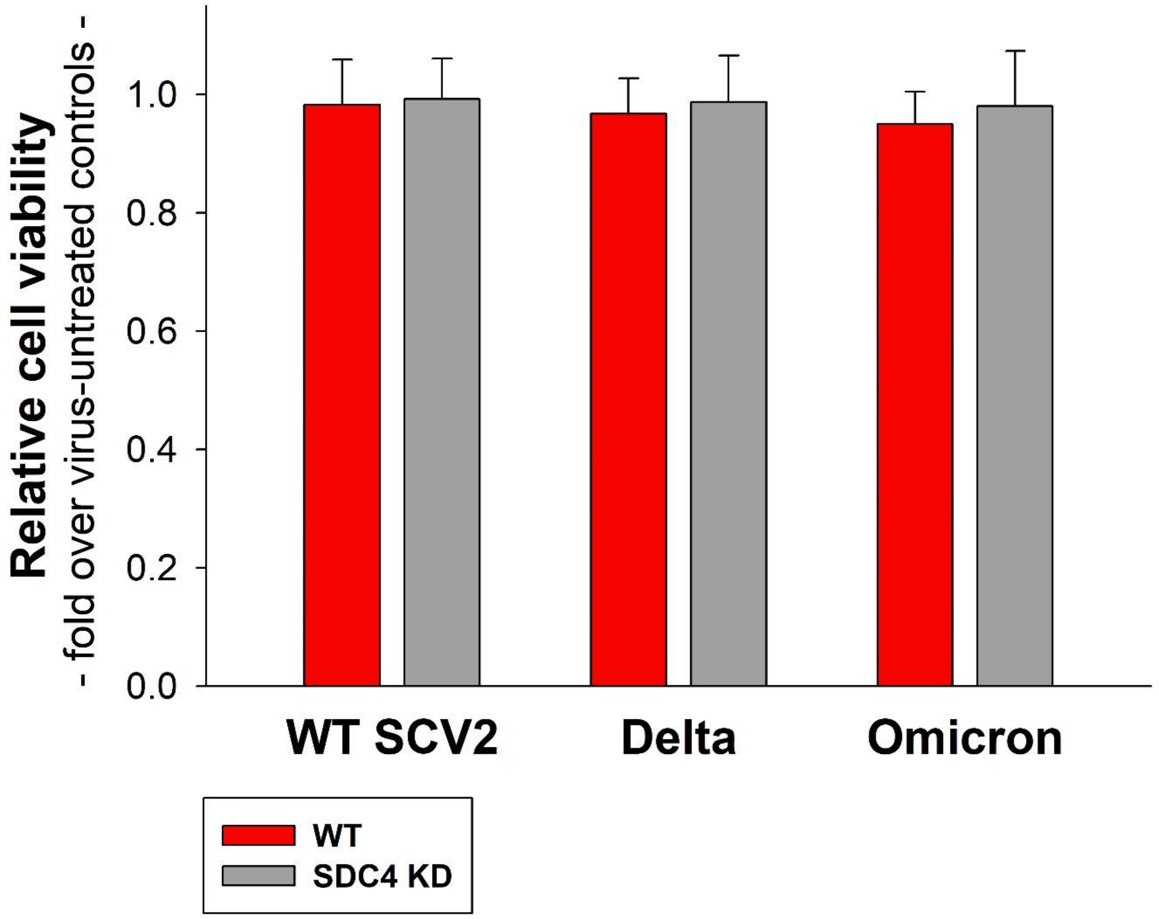

Figure S10. Heat-inactivated WT SCV2, Delta, and Omicron variants do not affect the cellular viability of WT Calu-3 cells. WT Calu-3 cells were incubated with either WT SCV2, Delta, or Omicron for 4 h at 1 MOI. Cellular viability was then measured with EZ4U assay and detected measures were then normalized to untreated cells as controls. The bars represent the mean + SEM of three independent experiments. Statistical significance vs. controls was assessed with ANOVA. Compared to controls, no statistically significant differences were detected in the viability of virus-treated cells.

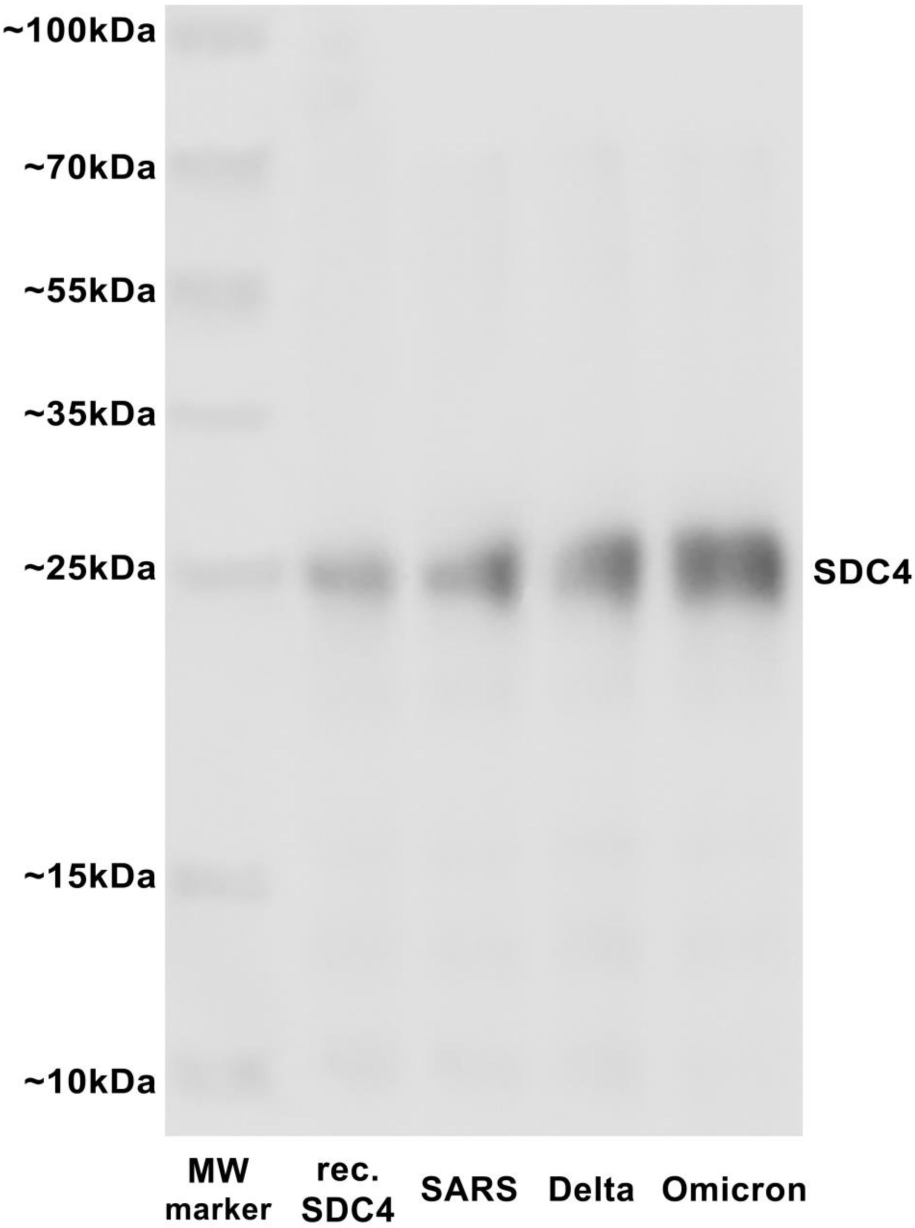

Figure S11. SDC4 binding of the WT SCV2 and the Delta, Omicron variants. SDS-PAGE showing SDC4 immunoprecipitated with an antibody specific for the spike's amino acid sequence 1000-1200 from extracts of virus-treated Calu-3 cells. Lanes 1: 1 ug of recombinant SDC4; Lanes 2–4: immunoprecipitates of Calu-3 cells treated with either WT SCV2, Delta, or Omicron, respectively. Standard protein size markers are indicated on the right. SDC4 signals were detected with UVITEC Alliance Q9 Advanced Imager, and the intensity of bands was analyzed with the NineAlliance© software.
